# Spatial and temporal axes impact ecology of the gut microbiome in juvenile European lobster (*Homarus gammarus*)

**DOI:** 10.1038/s41396-019-0546-1

**Published:** 2019-11-01

**Authors:** Corey C. Holt, Mark van der Giezen, Carly L. Daniels, Grant D. Stentiford, David Bass

**Affiliations:** 1 International Centre of Excellence for Aquatic Animal Health, Centre for Environment, Fisheries and Aquaculture Science (Cefas), Barrack Road, Weymouth, Dorset, DT4 8UB UK; 2 Biosciences, College of Life and Environmental Sciences, University of Exeter, Stocker Road, Exeter, UK; 3 The National Lobster Hatchery, South Quay, Padstow, UK; 4 The Centre for Sustainable Aquaculture Futures, College of Life and Environmental Sciences, University of Exeter, Stocker Road, Exeter, UK; 5 Department of Life Sciences, The Natural History Museum, Cromwell Road, Kensington, London, UK; 6 Present Address: Centre for Organelle Research, University of Stavanger, 4021 Stavanger, Norway

**Keywords:** Microbial ecology, Microbiome, Next-generation sequencing

## Abstract

Microbial communities within the gut can markedly impact host health and fitness. To what extent environmental influences affect the differential distribution of these microbial populations may therefore significantly impact the successful farming of the host. Using a sea-based container culture (SBCC) system for the on-growing of European lobster (*Homarus gammarus*), we tracked the bacterial gut microbiota over a 1-year period. We compared these communities with lobsters of the same cohort, retained in a land-based culture (LBC) system to assess the effects of the culture environment on gut bacterial assemblage and describe the phylogenetic structure of the microbiota to compare deterministic and stochastic assembly across both environments. Bacterial gut communities from SBCCs were generally more phylogenetically clustered, and therefore deterministically assembled, compared to those reared in land-based systems. Lobsters in SBCCs displayed significantly more species-rich and species-diverse gut microbiota compared to those retained in LBC. A reduction in the bacterial diversity of the gut was also associated with higher infection prevalence of the enteric viral pathogen Homarus gammarus nudivirus (HgNV). SBCCs may therefore benefit the overall health of the host by promoting the assembly of a more diverse gut bacterial community and reducing the susceptibility to disease.

## Introduction

The gut microbiome is a community of microorganisms that demonstrates complex interactions with both the host organism and within itself. Changes in microbiome structure can correlate with digestive enzyme activity and the subsequent pre-digestion of host ingesta. Consequently, the gut microbiota can aid in nutritional breakdown and contribute to the growth of the host [1–3]. A diverse microbiome can provide resistance against the proliferation of potentially pathogenic microbes, contributing to host immunity and improving survival [4, 5]. How the gut is colonised and maintained is somewhat unclear. However, considering its association with host processes, environmental determinants of gut community composition may subsequently impact growth and survival of the host [6–9].

The advent of high-throughput sequencing technologies and development of novel analytical approaches for profiling microbial communities has led to a rapid increase in studies of microbiomes and their interaction with their host organism [10–12]. Most gut microbiome studies focus on humans or other vertebrates [13]. There are relatively very few studies of aquatic invertebrate gut microbiomes as most invertebrate studies are limited to bees and other terrestrial insects [13], or economically important aquatic species such as penaeid shrimp. Anatomical and functional differences in the invertebrate digestive tract, compared to vertebrates, likely impose different influences on microbiome composition [14]. Furthermore, contrasting immune systems, i.e. the lack of adaptive immunity in invertebrates, may also impact bacterial colonisation of the gut along with host tolerance and retention of its commensals [15, 16]. Therefore, generalisations about vertebrate gut microbiomes may not reliably be extended to invertebrates. Invertebrates, however, are becoming increasingly important in modern-day aquaculture, comprising a multi-billion dollar global industry [17, 18]. Furthermore, poor gut health is an increasing issue for development of syndromic conditions which significantly reduce aquaculture production [18, 19].

With high market prices as a result of a relatively limited fishery, the European lobster (*Homarus gammarus*) has significant potential as a high value aquaculture species in Europe [20]. To aid stock enhancement and restocking of populations targeted by fisheries, hatchery-rearing of larval and early juvenile stages and their release to the fishery has been utilised as an approach to support European populations [21]. Given the relatively high cost of juvenile lobster production in land-based culture (LBC), the on-growing of juvenile life stages in so-called sea-based container culture (SBCC) systems has produced promising results in terms of growth and survival in recent years [22]. Sea-based container cultures are proposed to offer a reproducible and sustainable model for open sea rearing of lobsters given that once deployed, lobsters require relatively little management and, importantly, rely on naturally settled feed organisms in their diet [22].

Earlier studies on the gut microbiota of *H. gammarus* using Denaturing Gradient Gel Electrophoresis (DGGE) described a *Vibrio-*dominated community [5]. However, there have been no attempts to utilise high-throughput sequencing approaches to comprehensively characterise the gut microbiota of this economically important decapod. Analysis of faecal samples obtained from *H. gammarus* revealed significant changes in microbial composition between 6 and 12 months of age [23]. However, these communities were not analysed with respect to their taxonomic composition. Vibrionaceae and Pseudoalteromonadaceae were also the dominant inhabitants of the majority of spiny lobster (*Panulirus ornatus*) guts across different developmental life stages with mollicute sequences also accounting for a large proportion of the hindgut community sampled at the age of 13 months [24]. Temporal shifts in the dominance of Gammaproteobacteria and Mollicutes were also evident in gut communities isolated from Norway lobster (*Nephrops norvegicus*) [25]. However, to date, no comparisons in relation to culture environment have been made for any lobster species.

Here, we characterise the gut microbiota of juvenile European lobster over a one-year period, comparing a cohort retained in an LBC system with another originating in the land-based system but subsequently retained in SBCCs moored off the coastline of Cornwall, UK. We analyse the bacterial composition of the gut by comparing exact sequence variants (ESVs; [11]) derived from the bacterial V4 region of the ribosomal small-subunit (SSU) generated from individual animals, and use diversity indexes to compare the gut microbiomes of those individuals and the groups to which they belong. By assessing phylogenetic-based mean nearest taxon distance (MNTD), we test the role of deterministic assembly of the gut by analysing the phylogenetic relationships of its bacterial inhabitants. Finally, we compare the gut microbiome of healthy individuals with those displaying as histology-positive for the recently described Homarus gammarus nudivirus (HgNV), the first characterised clawed-lobster virus [26]. HgNV translocates through the gut to establish infection within cells of the associated hepatopancreas of its host.

## Methods

### Sample collection

Over the period of July 2016 to April 2017, 14,507 hatchery-reared juvenile lobsters were deployed in SBCCs anchored off the coast of Cornwall (St. Austell Bay 50°18.956N, 4°44.063W). The majority of those deployments (10,987 animals), including those used in the current study, occurred in the summer of 2016. Routine sampling (3, 6, 28, 39, 52, 104 weeks post deployment (WPD)) was carried out to monitor the incidence of disease in SBCC populations. In total, 1,698 animals were sampled over the 2-year period. A second set of lobsters (*n* = 400) from the same cohort were retained within the National Lobster Hatchery, Padstow, UK. Carapace length and survival were measured at each time point. Upon sampling, larger animals (39–104 WPD) were anaesthetised on ice prior to bisection through the dorsal line and the removal of the intestinal tract using sterile instruments. One half was fixed in Davidson’s Seawater fixative for histological processing, the other fixed in molecular grade ethanol for sequence analysis. Smaller animals (0–28 WPD) were fixed whole (in the above-mentioned fixatives) and underwent separate analyses. The gut was later aseptically removed using a dissecting microscope.

Twenty-four animals, representing a range of carapace lengths and two container types, were sequenced from each of the five sea-based time points up to and including 52 WPD. Owing to space constraints within the hatchery, 12 individuals from 0, 3, 39, and 52 WPD time points were chosen from the LBC group and sequenced. Nine individuals that had spent 104 weeks in LBC and suspected to be unwell were also sequenced.

### DNA extraction

DNA from individual guts was extracted using a CTAB/phenol:chloroform extraction method as described by Holt et al. [27]. Precipitated DNA was eluted in molecular grade water and quantified fluorometrically. DNA quality was assessed by measuring absorbance at 260 nm using a NanoDrop spectrophotometer (Thermo Scientific).

### Amplicon library preparation

Gut DNA was diluted to 1 ng/µL and transferred to two 96-well plates. Amplicon libraries were generated using the one-step custom PCR protocol and indexing primers described by Kozich et al. [12] and the 515fB (5′ GTGYCAGCMGCCGCGGTAA 3′) and 806rB (5′ GGACTACNVGGGTWTCTAAT 3′) V4 primers. All samples were amplified in triplicate in order to minimise PCR bias. Reactions were composed of 12.5 µL NEBNext PCR mix (New England BioLabs), 1.25 µL of both forward and reverse primers (10 µM), 7.5 µL of molecular grade water and 2.50 µL of template DNA (1 ng/µL). Initial denaturation was carried out at 98 °C for 30 s, followed by 30 cycles of 10 s denaturation at 98 °C, 30 s of annealing at 55 °C and 30 s of extension at 72 °C. Final extension was carried out at 72 °C for 2 min. Triplicate PCR reactions were then pooled prior to purification.

Amplicon libraries were purified with an Agenourt AMPure XP bead-based clean-up, in order to remove primer dimers and free primers. Cleaned DNA was resuspended in resuspension buffer (Illumina). Amplicon length was assessed using the D1000 ScreenTape system (Agilent). Expected fragment size was around 400 bp. Libraries were quantified using the Promega Glomax kit. To account for the low yield of some libraries, two separate library pools were made, diluted to 2 µM and mixed in accordance with the ratio of samples between them. The concentration of the final pool was determined using qPCR. One hundred and ninety-six libraries, including two controls, were sequenced using 250 bp reads (v2 chemistry) and the Illumina Miseq.

Raw reads were deposited in the NCBI sequence read archive under the BioProject PRJNA577421.

### Bioinformatics analysis

All reads were processed with the DADA2 analysis package in R [11]. Paired-end reads were trimmed according to visualised quality scores and DADA2’s standard filtering parameters: maxN = 2, truncQ = 2, rm.phix = TRUE, and maxEE = 2. DADA2’s parametric error model was fit using the first 100 million bases. Sequences were dereplicated and sequence variants inferred using the associated error model and pseudo-pooling. Filtered reads were merged and used to construct the amplicon sequence variant table. Denoised full length sequences were subsequently trimmed and chimeras removed. Taxonomy was assigned using the Silva database (v.132). Accuracy of the run was determined using a mock community of known samples, sequenced alongside a negative control. The negative control library contained no measurable DNA and produced less than 2% of sequences compared to the average read count.

All reads were BLASTed against the full nr database using the blastx function of DIAMOND v0.7.9 [28]. Classified reads were then visualised in MEGAN6 Community Edition v6.5.5 [29] and non-bacterial sequences were removed. NA taxonomic assignments were labelled with the lowest characterised taxonomic rank. Alpha diversity matrices were analysed within the phyloseq package [30]. ESVs were pruned prior to non-metric multidimensional scaling; ESVs that were not present in at least one sample were removed, as were samples that contained less than 1000 reads. Seed set at 2209.

Phyloseq and ggplot2 packages were used to visualise taxonomic profiles and diversity measures. The rgl package was used to visualise three-dimensional ordinations [31].

### Statistical analysis

Statistical analyses were conducted in the R statistical environment [32]. A series of linear models with interaction terms were used to correlate variation in the dataset. When comparing culture location, “day 0” samples were included within the “LBC” grouping. Permutational multivariate analysis of variance (PERMANOVA) was analysed using the adonis function of the vegan package. The same package was used to compute the multivariate homogeneity of group dispersions (Betadisper), both analyses were performed using 999 permutations [33].

### Phylogenetic analyses

Exact sequence variants from all individuals were aligned using the DECIPHER package in R [34]. A maximum likelihood phylogenetic tree inference was constructed with a generalised time-reversible model with gamma rate variation using the phangorn package [35] and subsequently used to calculate phylogenetic structuring.

### Phylogenetic community structure

Mean nearest taxon index (MNTD) computes the mean of the phylogenetic distance between an ESV in a given community and its closest relative within that sample. The standard effect size of phylogenetic community structure (ses.MNTD) compares the divergence away from a random, null model of distribution, measured in standard deviations, which can then be used to assess assemblage of the community as a reflection of their phylogenetic relationship. For individual samples, a ses.MNTD value of > −2 and < 2 indicates that coexisting taxa are no more related than expected by chance. Values > 2 indicate phylogenetic overdispersion and taxa are more distantly related than the null model. Values < −2 indicate phylogenetic clustering and taxa are more closely related than expected by chance. A mean ses.MNTD value representing multiple communities that is significantly greater or less than 0 is said to represent phylogenetic overdispersion and clustering respectively. ses.MNTD values are equivalent to the negative of the nearest-taxon index (NTI) and were computed using the picante package with null model = “taxa.names”, abundance.weighted = FALSE and 999 random permutations.

### Molecular confirmation of viral infection

The HgNV_DNAPol_F1: 5′ACTTGAAGCTGTGCGTGACT 3′ and HgNV_DNAPol_R1: 5′ TGTATGTCTTGCGGCCCATT 3′ diagnostic primer set was used to confirm viral infection in HP and gut tissues of 104 LBC animals. PCR reactions and thermal cycler settings were as described in Holt et al. [26].

## Results

### Temporal and spatial changes affect bacterial profiles of the lobster gut

A total of 7,928,959 bacterial SSU V4 region reads from 183 samples remained after filtering. On average, each sample was represented by 43,328 ± 1529 reads. Sequencing depth ranged from 2,698–148,629 reads across all samples. Good’s coverage index exceeded 0.99 in all filtered samples, indicating less than 1% of reads in each sample only appear once in that sample (Supplementary Fig. 1A) and rarefaction curves indicated near-saturation of community coverage (Supplementary Fig. 1B). It should be noted, however, in a bid to remove artefactual sequences, singletons were only retained if they were present in multiple samples.

**Fig. 1:**
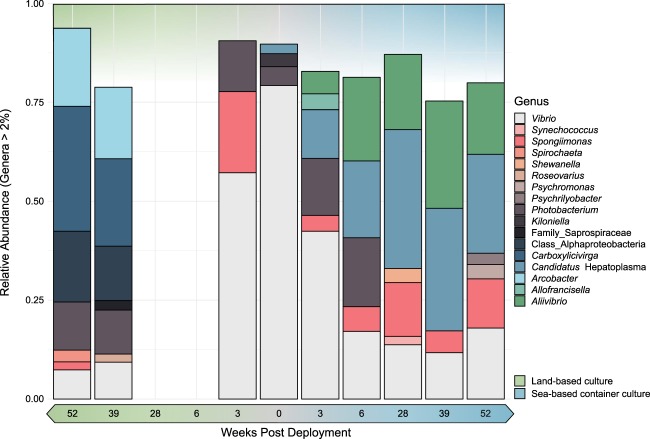
Average bacterial profiles of all animals sampled over 52 weeks. Bacterial genera representing more than 2% of entire 16S community. Genera coloured according to key. Time increases towards to extremities of the *x* axis from the pre-deployment control (0 PDC) at the centre. Green = land-based culture (LBC). Blue = sea-based container culture (SBCC)

The average profile of a 0-week pre-deployment control (PDC) individual was composed of 96 ESVs comprising four bacterial genera with over 2% relative abundance. *Vibrio* spp. dominated this community (58 ESVs), followed by *Photobacteria* (33 ESVs)*, Kiloniella* (1 ESV) and “*Candidatus* Hepatoplasma” (four ESVs) (Fig. 1, Table 1, Supplementary Table 1). The latter was not detected in average profiles at 3, 39, and 52 WPD in the LBC system but made up substantial proportions of all SBCC group profiles post deployment. Eighteen ESVs belonging to this assignment were shared across all SBCC profiles. Sequence variants aligned with “*Candidatus* Hepatoplasma” in the Silva database with relatively low identity, ranging from 78.7% to 90.1%. *Vibrio* spp. continued to comprise significant proportions of the guts sampled at all subsequent time points, but with a general decline in relative abundance over time. With the exception of the 0 PDC group, the number of ESVs attributed to *Vibrio* relatives at each time point, however, was relatively constant (Table 1). The *Aliivibrio* genus was first detected 3 weeks after deployment, and then constituted an average of 22 ± 2% of the community make-up for the rest of the sampled period (Supplementary Table 1). *Spongiimonas* was also present in all sea-based group profiles, in addition to 3- and 52-week LBC groups. Conversely, *Photobacterium* lineages were detected in all hatchery group profiles, in addition to 3- and 6-week SBCC groups, which included considerably more sequence variants compared to LBC groups (Table 1). A single *Carboxylicivirga* sequence and four shared *Arcobacter* ESVs made up substantial proportions of the 39- and 52-week LBC groups.

**Table 1:** Exact sequence variant count of bacterial genera representing more than 2% relative abundance

Genus	Number of ESVs										
	52 LBC	39 LBC	28 LPC	6 LBC	3 LBC	0 PDC	3 SBCC	6 SBCC	28 SBCC	39 SBCC	52 SBCC
*Vibrio*	17	22	–	–	15	58	33	32	32	23	30
*Synechococcus*	–	–	–	–	–	–	–	–	6	–	–
*Spongiimonas*	1	–	–	–	1	–	2	2	4	3	3
*Spirochaeta*	2	–	–	–	–	–	–	–	–	–	–
*Shewanella*	–	–	–	–	–	–	–	–	15	–	–
*Roseovarius*	–	7	–	–	–	–	–	–	–	–	–
*Psychromonas*	–	–	–	–	–	–	–	–	–	–	14
*Psychrilyobacter*	–	–	–	–	–	–	–	–	–	–	2
*Photobacterium*	2	2	–	–	3	33	10	11	–	–	–
*Kiloniella*	–	–	–	–	–	1	–	–	–	–	–
Family_Saprospiraceae	–	44	–	–	–	–	–	–	–	–	–
Class_Alphaproteobacteria	9	20	–	–	–	–	–	–	–	–	–
*Carboxylicivirga*	1	1	–	–	–	–	–	–	–	–	–
*Candidatus* Hepatoplasma	–	–	–	–	–	4	6	7	10	8	9
*Arcobacter*	3	2	–	–	–	–	–	–	–	–	–
*Allofrancisella*	–	–	–	–	–	–	8	–	–	–	–
*Aliivibrio*	–	–	–	–	–	–	8	7	13	6	9

Several genera were limited to one or more time point and only *Vibrio* spp. were isolated from all sample groups, regardless of culture environment (Fig. 1). Two ESVs were not assigned a taxonomy by the analysis pipeline. Manual classification of these sequences later resolved their identity. The most abundant unclassified ESV from the 39 and 52 LBC groups corresponded to a genus of Sphingomonadaceae (Class_Aphaproteobactera). The remaining ESV making up the 39LBC group were assigned as an uncultured Saprospiraceae.

**Fig. 2:**
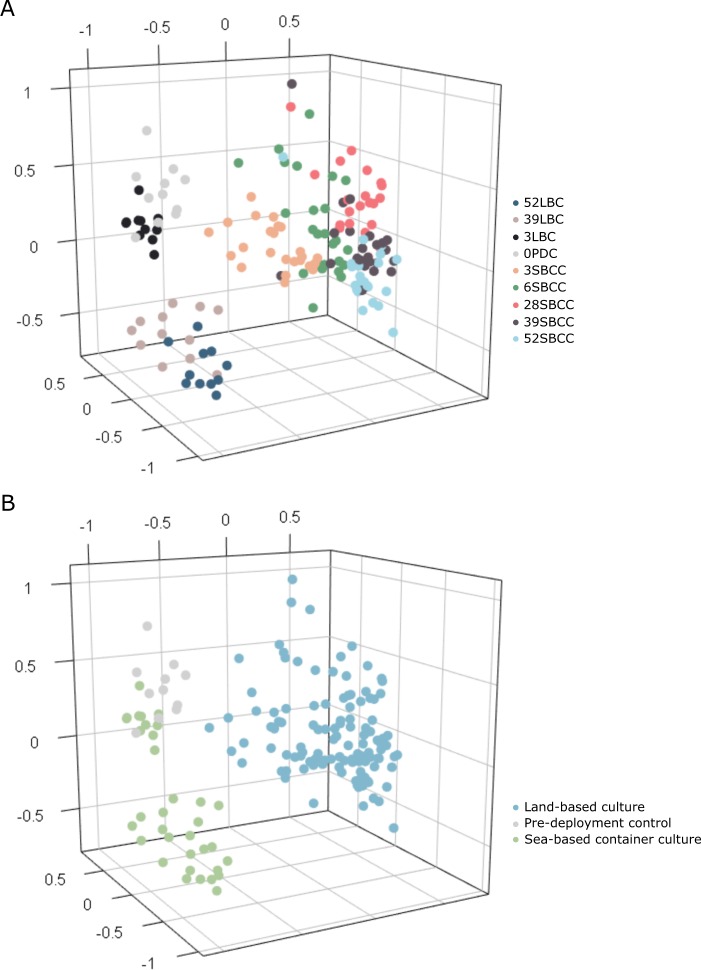
Three-dimensional non-metric multidimensional scaling (NMDS) of all gut samples. Unweighted non-metric multidimensional scaling (NMDS) using the Bray-Curtis measure of dissimilarity over three axes. Stress = 0.130. **a** Coloured according to sample group. **b** Coloured according to culture environment

Non-metric multidimensional scaling shows that all samples clustered according to group, defined by age and culture environment (Fig. 2a; stress: 0.130). A corresponding stressplot indicates the non-metric fit (*R*^2^) of the ordination distance to the observed dissimilarity was 0.983 (Supplementary Fig. 2). Centroid analysis of groups within the ordination demonstrates observed clustering was statistically significant (PERMANOVA, *p* < 0.001). Pairwise analysis showed that all groups were significantly different from each other (PEMRANOVA, *p* ≤ 0.002), with the exception to the 39–52 LBC comparison (PERMANOVA, *p* = 0.223). Dispersion of samples within clusters, i.e. variation within each group, was also significant (Betadispersion, *p* < 0.001). The same ordination grouped by culture environment alone shows that LBC and SBCC clusters were significantly distinct (*p* < 0.001; Fig. 2b). However, variation between samples within environments is not significant (*p* = 0.921), suggesting centroid analysis of clusters representing culture environment is not confounded by differential rates of dispersion, i.e., the sample variation within each location is comparable.

**Fig. 3:**
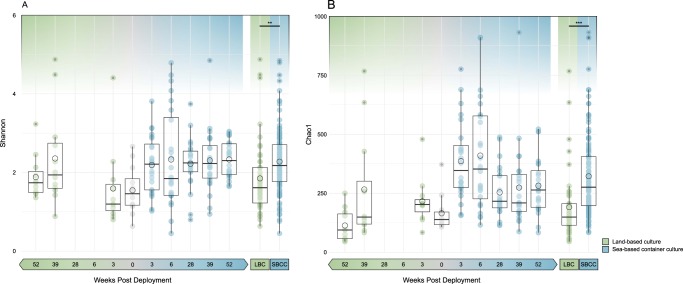
Alpha diversity measures of all sample groups. **a** Shannon’s measure of species diversity across all sample groups. **b** Chao1 estimate of species richness across all sample groups. Green = land-based culture (LBC). Blue = sea-based container culture (SBCC). Environmental comparison “LBC” (including day 0) and “SBCC” represent combined data of all corresponding groups. Boxes labelled with groups that are significantly different. **p < =0.01, ***p < =0.001

The averages of both species richness (Chao1) and species diversity (Shannon’s diversity) of the gut were significantly higher in lobsters from SBCC systems compared to LBC (Fig. 3. Chao1; *p*-value < 0.001. Shannon’s; *p*-value = 0.004). The progression of time in LBC did not correlate to any significant changes in bacterial richness or diversity, according to the linear model. However, in SBCC, there was a significant reduction in species richness with time after deployment (*p*-value < 0.001). Bacterial diversity remained relatively constant in SBCC. It should be noted that, according to the linear model, culture environment does not explain all of the variability found in the data.

### Deterministic processes impact gut assembly in SBCC

**Fig. 4:**
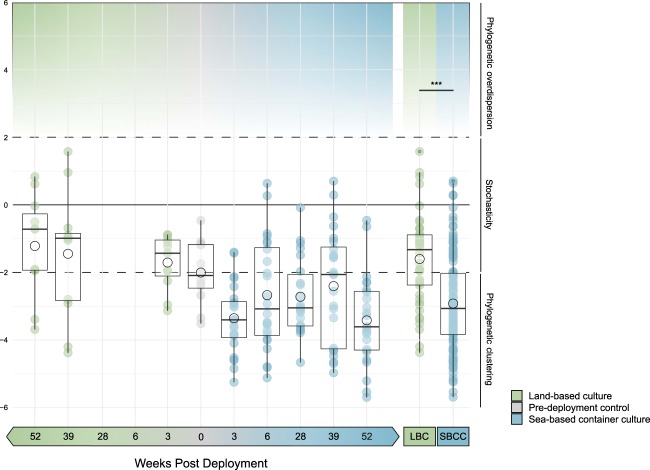
Standard effect size of mean-nearest-taxon index (ses.MNTD) indicating phylogenetic clustering of sequence variants. Standard deviation of mean nearest taxon index (MNTD) from random model. Ses.MNTD values >2 indicate phylogenetic overdispersion of taxa, 2 < & > −2 indicate stochastic distribution across phylogeny, < −2 indicate phylogenetic clustering. Green = land-based culture (LBC). Blue = sea-based container culture (SBCC). Environmental comparison “LBC” (including day 0) and “SBCC” represent combined data of all corresponding groups. Boxes labelled with groups that are significantly different. ***p < =0.001

The ses.MNTD representing the 0 WPD control group was −2.091 ± 0.268, indicating that bacterial taxa within these animals are on the border between random distribution and phylogenetic clustering (Fig. 4). The average ses.MNTD value for remaining LBC groups remain within the limits of implicit stochasticity (−2 > *x* < 2) and become more indicative of random assemblage (i.e., the null model) as time increases from 3 WPD to 52 WPD (3 LBC = −1.432 ± 0.244, 39 LBC = −0.991 ± 0.528, 52 LBC = −0.721 ± 0.450) (Fig. 4). Average ses.MNTD values for all SBCC groups are less than −2 implying a greater degree of phylogenetic clustering of bacteria and deterministic assembly. The degree of phylogenetic clustering, however, does not correlate with an increase in the age of the sample group (3 SBCC = −3.403 ± 0.209, 6 SBCC = −3.076 ± 0.331, 28 SBCC = −3.047 ± 0.241, 39 SBCC = −2.061 ± 0.344, 52 SBCC = −3.608 ± 0.272) (Fig. 4). Overall, bacterial colonisers of SBCC lobster guts are significantly more phylogenetically clustered compared to those in LBC (*p* value < 0.001).

### The presence of an enteric virus correlates with changes to the bacterial gut microbiome

**Fig. 5:**
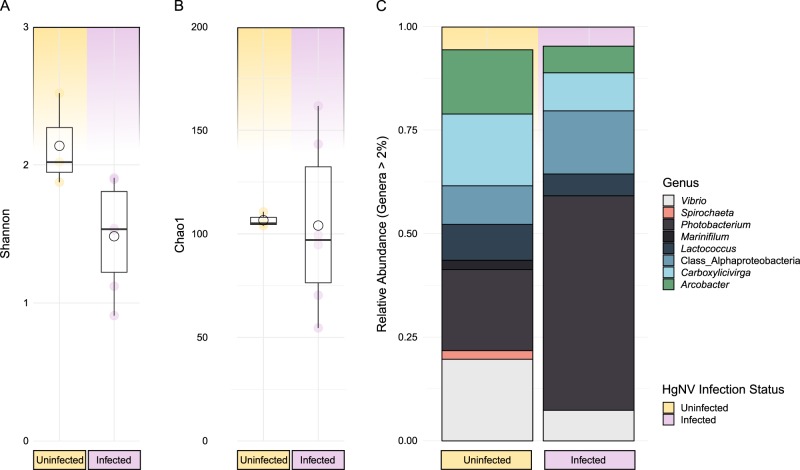
Changes to gut microbiota in the presence of Homarus gammarus nudivirus (HgNV). **a** Shannon’s measure of species diversity across healthy and infected individuals sampled at 104 weeks. **b** Chao1 estimate of species richness. **c** Bacterial genera representing more than 2% of entire 16S community. Yellow = HgNV-negative samples (*n* = 3). Pink = HgNV-positive samples (*n* = 6). Genera coloured according to key

Histological analysis of a group of animals that had spent 104 weeks in LBC showed intranuclear inclusions, a characteristic sign of viral infection, in the HP of six out of the nine animals. PCR amplification of the viral DNA polymerase gene of the recently characterised nudivirus, HgNV [26] produced positive signal for the virus in all six HP tissue samples. Individuals infected with HgNV harboured a less diverse bacterial gut microbiota compared to uninfected lobsters (Fig. 5a). Furthermore, gut bacterial richness of infected individuals was more variable than those tested negative for HgNV infection (Fig. 5b). Although there are compositional differences when comparing the average profiles of infected vs uninfected animals, for example, the genera *Marinifilum* and *Spirochaeta* are present in the gut of uninfected animals, but not in virus-infected animals (Fig. 5c), there are not any clear associations when comparing individuals. Generally, however *Photobacterium* spp. are consistently more dominant in virus-infected animals (Fig. 5c). The unassigned lineage again corresponds to the uncultured Alphaproteobacteria isolated likely belonging to the Sphingomonadaceae.

## Discussion

Our results highlight the high degree of plasticity of the gut microbiota of the European lobster and demonstrate how environment, age (during early life), and infection status with a specific virus can correlate with differences in bacterial community composition. Individuals raised in SBCCs were associated with a more diverse gut microbiome, which may confer subsequent benefits to the health and growth of their hosts. Rearing lobsters in a more microbially rich and diverse natural marine environment, as opposed to a land-based system, likely encourages the selection and colonisation of a more diverse gut community. Therefore, the SBCC could potentially benefit production of this and other species.

Biological community assembly can be governed by both stochastic and deterministic processes [36–39]. Stochastic processes include those pertaining to passive dispersal and ecological drift, i.e. random loss and gain, whereas deterministic colonisation refers to environmental selection governed by the relative differences in ecological fitness of its inhabitants [39, 40]. Phylogenetic clustering of a bacterial community indicates a greater degree of environmental filtering and a non-random association with its environment, in this case the gut, as closely related species are predicted to be more ecologically similar and therefore subject to a greater degree of competition. There are conflicting results as to how important deterministic processes are in the establishment of gut communities in zebrafish of increasing age [40, 41] yet we did not observe any temporal trends in lobsters held in either LBC or SBCCs. However, 52 weeks is a relatively short proportion of the typical life span of a healthy lobster. Our results suggest that LBC animals generally relied on stochastic means of gut assembly throughout their development indicating that random dispersal of potential bacteria colonisers can account for considerable variations in gut community. Indeed, variation within LBC groups is greater than sea-based groups at corresponding time points, and stochasticity has been demonstrated to induce heterogeneity in bacterial gut samples of *Caenorhabditis elegans* [42]. Sea-based lobster gut samples typically harboured a more phylogenetically clustered bacterial community compared to LBC animals of the same age, suggesting that gut communities of SBCC lobsters were more deterministically assembled; i.e., there are more factors limiting the random assemblage of bacteria in the gut. Previous studies have shown that the invertebrate gut microbiota tends to be distinct from that of the host rearing water [1, 43–45]. The gut and its ingested substrates may therefore support the positive selection of relatively rare bacterial lineages from the complex surrounding water column. Despite the majority of sea-based samples from each of the time points indicating phylogenetic clustering, many corresponded to ses.MNTD values of greater than −2, therefore the degree of environmental filtering is likely influenced by individual traits which vary within a population; such as growth capacity [3] or health state of the host [46]. It is also worth noting that animals were sampled in a random manner with respect to moulting stage. The moult cycle may impact the presence of specific microbes and therefore contribute to inter-sample variability [47].

The *Vibrio* genus, belonging to the phylum Proteobacteria, is commonly reported as the dominant genus of invertebrate digestive tracts [1] and is ubiquitous within many water column samples [48]. Several *Vibrio* spp. are infamous for causing disease in humans, however, many also pose risks to marine invertebrates. *Vibrio harveyi*, for example, can infect and disrupt the epidermal tissue of the digestive tract and can limit the production of penaeid shrimp [49], *Vibrio parahaemolyticus* encoding toxic *Photorhabdus* insect-related binary toxins can cause acute hepatopancreatic necrosis disease (AHPND) and result in large production losses in shrimp aquaculture [50] and *Vibrio owensii* DY05 can cause mass mortalities of ornate spiny lobster (*P. ornatus*) [51]. Many species, however, are commensal and thought to be opportunistic in their nature [52]. Non-pathogenic strains may have the potential to be employed as a probiotic. The addition of both *Vibrio alginolyticus* and *Vibrio gazogenes*, have resulted in a reduction of several pathogenic *Vibrio* spp. in the guts of *Litopenaeus vannamei* [53]*. Vibrio* spp. may also confer benefits by producing extracellular chitinases [54, 55], which could aid in the digestion of prey and could also breakdown the host exuvia after ecdysis, routinely ingested to promote calcification of the new carapace. The *Aliivibrio* genus, erected to differentiate *A. fischeri* from other *Vibrio*, contains mainly salmonid pathogens associated with low water temperatures [56]. It should be noted that the V4 region of the rRNA SSU is not capable of fully differentiating between different species of the *Vibrio* and *Aliivibrio* genera as numerous database entries for both genera share 100% sequence identity.

*“Candidatus* Hepatoplasma crinochetorum” is a monophyletic species of Mollicutes first isolated from the hepatopancreas of the terrestrial isopod, *Porcellio scaber* [57]. The presence of the symbiont in isopods was positively correlated with survival on low-quality food suggesting a beneficial endosymbiosis between the two organisms [58]. As terrestrial isopods feed on low-nutrient, decaying plant matter, an association from which they can better sequester nutrition from their ingesta should be beneficial. It is hypothesised that symbiotic relationships such as this may have facilitated the expansion of isopods to terrestrial environments as no such bacteria were found in the hepatopancreas of isopods from the marine environment [59]. An ESV representative of the “*Candidatus* Hepatoplasma” assignment, detected in lobster, was identical to that isolated from Norway lobster (*Nephrops nervegicus*) [45] and 96% identical to a clone isolated from the high intertidal/sublittoral isopod *Ligia occidentalis* [60].

*Spongiimonas* is a Gram-negative, aerobic genus within Flavobacteriaceae that has been isolated from a marine sponge [61]. Our ESVs annotated as *Spongiimonas* are equally similar to several uncultured bacteria isolated from the guts of *N. norvegicus* [45]. Flavobacteriaceae is a large family of Bacteroidetes, many of which are responsible for several important fish diseases [62]. Flavobacteriaceae have been isolated from lesions of lobsters infected with epizootic shell disease (ESD), a cuticular disease causing erosion of the carapace in American lobster (*Homarus americanus*) [63]. Although the exact etiological agent(s) of ESD are unknown, and wild European lobsters seem to be unaffected by this disease, American lobsters displaying signs of ESD have been found in Norwegian waters [64] and phenotypic signs of the infection can make the animal unmarketable.

The *Carboxylicivirga* and *Arcobacter* genera make up substantial proportions of 39- and 52-week LBC animals. The ESV assigned to *Carboxylicivirga* is highly similar to *Roseobacter* clones associated with harmful algal blooms (KY277569 and KY277241) and those found in the gut of *N. norvegicus* and the mud crab *Scylla paramamosain* [65]. However very little information is known about the role of this relatively newly recognised genus in the environment or in any host species from which it has been detected [66]. Several *Arcobacter* species have been detected from both the marine environment [67, 68] and shellfish samples [69–72]. Furthermore, several species are recognised as emerging human pathogens and can be associated with gastrointestinal disease [73, 74]. The ESV assigned to *Arcobacter* isolated from lobster guts are identical to those isolated from the guts of abalone (LC180340), sea cucumber (JX170271) as well as sequences isolated from the water column itself (GU584643 and EU142059).

The unclassified ESVs limited to the LBC system and assigned to Sphingomonadaceae and Saprospiraceae could both represent biofouling species derived from the recirculating system. Sphingomonadaceae, a family of Alphaproteobacteria, have been identified in the guts of oriental river prawn (*Macrobrachium nipponense)* [75] but also isolated from fouled membranes of water filtration systems [76], and Saprospiraceae, a family of Bacteroidetes, have been isolated from shrimp rearing water [77, 78] and recirculating systems [79, 80] likely explaining their association with hatchery individuals. The Sphingomonadaceae ESV was also identical to a sequence derived from the guts of reared *N. norvegicus* (JN092211) [45].

In addition to direct causal links between particular bacterial species and disease, several studies claim that changes in bacterial diversity of the gut correlates to host health and the incidence of, particularly enteric, disease [81]. Bacteria within the lumen of the gut may contribute to host health in several ways; (1) as attachment sites within the gut are ultimately finite, the presence of a commensal community may limit the colonisation and subsequent proliferation of potentially pathogenic microbes, in a process described as colonisation resistance [4]; (2) through the production of antimicrobial peptides, members of this community can subsequently affect the abundance of other colonisers and therefore have the potential to antagonise pathogens [82]; (3) by stimulating the host immune system, this community can influence host tolerance to other microbes in the gut [83]. Antagonistic potential within a diverse gut perhaps increases the chances of resistance to a new pathogen and could reduce the susceptibility to incoming pathogens, preventing the establishment of infection. A reduction in diversity and subsequent compromise to colonisation resistance and its inherent redundancy could allow the proliferation of enteric pathogens such as HgNV. Although HgNV replicates in the nuclei of the hepatopancreatic epithelial cells, nudiviruses can colonise the host via the digestive tract, relying on entry through the intestinal epithelia. A lower prevalence of the virus was detected in SBCC animals compared to LBC control groups [26]. A possible explanation for this is that sea-based animals have a more diverse gut microbiome and the incidence of viral disease is dependent on the degree of the gut’s resistance to its colonisation and subsequent infection. It should be noted however that sample size of infected versus healthy individuals was low (*n* = 9), and this should be treated as preliminary data. Furthermore, we cannot distinguish between cause or effect within this infection model. Alternatively, an infection such as HgNV and associated compromise to host immunity may lead to a reduction in host selection pressures within the gut and lead to the observed variations in richness and diversity of the microbiota. Previous studies have indicated that a disease state may lessen the importance of deterministic assembly of the gut microbiota and instead induce stochasticity as trade-offs divert resources to immune function and other host processes [84]. Shrimp infected with AHPND demonstrate more stochastic means of assembly than healthy animals of the same age [46, 85]. Possibly because of the small sample size of HgNV-infected individuals at distinct time points, we did not observe significant differences in ses.MNTD values corresponding to infection state.

As a knock-on effect of disease inducing differential abundance of the microbiota, if particular taxa in the microbiome are more adept at nutritional breakdown, there may be subsequent effects on the growth of the host which detriment production. Penaeus monodon nudivirus branches as a sister lineage to HgNV and has been noted to suppress growth rates in aquaculture [86]. Experimental designs utilising gnotobiotic organisms, or those with a predefined microbiota, may help clarify these complex interactions and may discern between cause and effect. We indeed observed significant size variation between lobsters of the same age. Although genetic variation and differential food intake was not controlled in this experiment, we hypothesised that size variation in cohabiting animals could be influenced by individual variation of the gut microbiota and its ability to utilise available foodstuff, as keystone bacterial taxa are associated with digestive enzyme activity and growth of the host [87]. However, there were no significant variations in bacterial richness and diversity when comparing different sized animals of the same age, or indeed all samples after age-discriminatory taxa were predicted with a random forest model and removed from the entire dataset.

More samples are needed to analyse HgNV infection in relation to microbiome depletion and to test the significance of these suggested differences. If HgNV colonisation and infection is dependent on microbes in the gut, variability of the gut microbiota in early stage animals may account for the differential ability of HgNV to infect individuals within a population and subsequently influence its abundance in older animals. If this is the case, the seeding of bacteria within the gut of juvenile lobsters (e.g. by ensuring they are fed a diverse bacterial diet) in a land-based system, or preconditioning exposure to the natural environment prior to release, could facilitate the establishment of more robust and/or healthy gut in later life stages. The application of metagenomic and/or transcriptomic analysis will further aid in elucidating the functional potential of the European lobster gut and any environmental-dependent impacts on metabolic processes of the host. Together, this information could be used in the design of novel and appropriate probiotic supplements to better cultivate this species.

## Supplementary information

Supplementary Information

Supplementary Table 1

Supplementary Figure 1

Supplementary Figure 2
