# Supplementary material for: Spatial and temporal axes impact ecology of the gut microbiome in juvenile European lobster (Homarus gammarus)
Source: ISME J. 2019 Nov 1;14(2):531–43. doi: 10.1038/s41396-019-0546-1 (PMC6976562; doi:10.1038/s41396-019-0546-1)
Supplement: Supplementary file 1 — Supplementary Information [file 41396_2019_546_MOESM1_ESM.docx]

**Spatial and temporal axes impact ecology of the gut microbiome in juvenile European lobster *(Homarus gammarus)***

*Corey C. Holt^a#,b,c,d^, Mark van der Giezen^b,d^, Carly L. Daniels^c^, Grant D. Stentiford^a,d^ and David Bass^a,d,e^*

**Supplementary Figure 1. Sequencing coverage across all samples.**

Estimations of community saturation across all individuals. A: Good’s coverage estimates. B: Rarefaction of increasing sequencing effort. Plots coloured according to sample group.

**Supplementary Figure 2. Shepard plot indicating fit of NMDS**

Shepard plot indicating fit of observed dissimilarity to ordination distance

**Supplementary Table 1. Percentage abundances from average bacterial profiles of all animals sampled over 52 weeks.**
