# Supplementary material for: Spatial and temporal axes impact ecology of the gut microbiome in juvenile European lobster (Homarus gammarus)
Source: ISME J. 2019 Nov 1;14(2):531–43. doi: 10.1038/s41396-019-0546-1 (PMC6976562; doi:10.1038/s41396-019-0546-1)
Supplement: Supplementary file 2 — Supplementary Table 1 [file 41396_2019_546_MOESM2_ESM.docx]

| **Sample Group** | **Abundance** | **Phylum** | **Class** | **Order** | **Family** | **Genus** |
| --- | --- | --- | --- | --- | --- | --- |
| 39SBCC | 0.27 | Proteobacteria | Gammaproteobacteria | Vibrionales | Vibrionaceae | Aliivibrio |
| 6SBCC | 0.21 | Proteobacteria | Gammaproteobacteria | Vibrionales | Vibrionaceae | Aliivibrio |
| 28SBCC | 0.19 | Proteobacteria | Gammaproteobacteria | Vibrionales | Vibrionaceae | Aliivibrio |
| 52SBCC | 0.18 | Proteobacteria | Gammaproteobacteria | Vibrionales | Vibrionaceae | Aliivibrio |
| 3SBCC | 0.06 | Proteobacteria | Gammaproteobacteria | Vibrionales | Vibrionaceae | Aliivibrio |
| 3SBCC | 0.04 | Proteobacteria | Gammaproteobacteria | Francisellales | Francisellaceae | Allofrancisella |
| 52LBC | 0.2 | Epsilonbacteraeota | Campylobacteria | Campylobacterales | Arcobacteraceae | Arcobacter |
| 39LBC | 0.18 | Epsilonbacteraeota | Campylobacteria | Campylobacterales | Arcobacteraceae | Arcobacter |
| 28SBCC | 0.35 | Tenericutes | Mollicutes | Entomoplasmatales | Entomoplasmatales_  Incertae_Sedis | Candidatus_Hepatoplasma |
| 39SBCC | 0.31 | Tenericutes | Mollicutes | Entomoplasmatales | Entomoplasmatales_  Incertae_Sedis | Candidatus_Hepatoplasma |
| 52SBCC | 0.25 | Tenericutes | Mollicutes | Entomoplasmatales | Entomoplasmatales_  Incertae_Sedis | Candidatus_Hepatoplasma |
| 6SBCC | 0.19 | Tenericutes | Mollicutes | Entomoplasmatales | Entomoplasmatales_  Incertae_Sedis | Candidatus_Hepatoplasma |
| 3SBCC | 0.12 | Tenericutes | Mollicutes | Entomoplasmatales | Entomoplasmatales_  Incertae_Sedis | Candidatus_Hepatoplasma |
| 0LBC | 0.02 | Tenericutes | Mollicutes | Entomoplasmatales | Entomoplasmatales_  Incertae_Sedis | Candidatus_Hepatoplasma |
| 52LBC | 0.32 | Bacteroidetes | Bacteroidia | Bacteroidales | Marinilabiliaceae | Carboxylicivirga |
| 39LBC | 0.22 | Bacteroidetes | Bacteroidia | Bacteroidales | Marinilabiliaceae | Carboxylicivirga |
| 52LBC | 0.18 | Proteobacteria | Alphaproteobacteria | Class_Alphaproteobacteria | Class_Alphaproteobacteria | Class_Alphaproteobacteria |
| 39LBC | 0.14 | Proteobacteria | Alphaproteobacteria | Class_Alphaproteobacteria | Class_Alphaproteobacteria | Class_Alphaproteobacteria |
| 39LBC | 0.02 | Bacteroidetes | Bacteroidia | Chitinophagales | Saprospiraceae | Family_Saprospiraceae |
| 0LBC | 0.03 | Proteobacteria | Alphaproteobacteria | Rhodovibrionales | Kiloniellaceae | Kiloniella |
| 6SBCC | 0.17 | Proteobacteria | Gammaproteobacteria | Vibrionales | Vibrionaceae | Photobacterium |
| 3SBCC | 0.14 | Proteobacteria | Gammaproteobacteria | Vibrionales | Vibrionaceae | Photobacterium |
| 3LBC | 0.13 | Proteobacteria | Gammaproteobacteria | Vibrionales | Vibrionaceae | Photobacterium |
| 52LBC | 0.12 | Proteobacteria | Gammaproteobacteria | Vibrionales | Vibrionaceae | Photobacterium |
| 39LBC | 0.11 | Proteobacteria | Gammaproteobacteria | Vibrionales | Vibrionaceae | Photobacterium |
| 0LBC | 0.05 | Proteobacteria | Gammaproteobacteria | Vibrionales | Vibrionaceae | Photobacterium |
| 52SBCC | 0.03 | Fusobacteria | Fusobacteriia | Fusobacteriales | Fusobacteriaceae | Psychrilyobacter |
| 52SBCC | 0.04 | Proteobacteria | Gammaproteobacteria | Alteromonadales | Psychromonadaceae | Psychromonas |
| 39LBC | 0.02 | Proteobacteria | Alphaproteobacteria | Rhodobacterales | Rhodobacteraceae | Roseovarius |
| 28SBCC | 0.04 | Proteobacteria | Gammaproteobacteria | Alteromonadales | Shewanellaceae | Shewanella |
| 52LBC | 0.03 | Spirochaetes | Spirochaetia | Spirochaetales | Spirochaetaceae | Spirochaeta |
| 3LBC | 0.2 | Bacteroidetes | Bacteroidia | Flavobacteriales | Flavobacteriaceae | Spongiimonas |
| 28SBCC | 0.14 | Bacteroidetes | Bacteroidia | Flavobacteriales | Flavobacteriaceae | Spongiimonas |
| 52SBCC | 0.12 | Bacteroidetes | Bacteroidia | Flavobacteriales | Flavobacteriaceae | Spongiimonas |
| 6SBCC | 0.06 | Bacteroidetes | Bacteroidia | Flavobacteriales | Flavobacteriaceae | Spongiimonas |
| 39SBCC | 0.05 | Bacteroidetes | Bacteroidia | Flavobacteriales | Flavobacteriaceae | Spongiimonas |
| 3SBCC | 0.04 | Bacteroidetes | Bacteroidia | Flavobacteriales | Flavobacteriaceae | Spongiimonas |
| 52LBC | 0.02 | Bacteroidetes | Bacteroidia | Flavobacteriales | Flavobacteriaceae | Spongiimonas |
| 28SBCC | 0.02 | Cyanobacteria | Oxyphotobacteria | Synechococcales | Cyanobiaceae | Synechococcus |
| 0LBC | 0.79 | Proteobacteria | Gammaproteobacteria | Vibrionales | Vibrionaceae | Vibrio |
| 52SBCC | 0.18 | Proteobacteria | Gammaproteobacteria | Vibrionales | Vibrionaceae | Vibrio |
| 3SBCC | 0.42 | Proteobacteria | Gammaproteobacteria | Vibrionales | Vibrionaceae | Vibrio |
| 28SBCC | 0.14 | Proteobacteria | Gammaproteobacteria | Vibrionales | Vibrionaceae | Vibrio |
| 39SBCC | 0.12 | Proteobacteria | Gammaproteobacteria | Vibrionales | Vibrionaceae | Vibrio |
| 39LBC | 0.09 | Proteobacteria | Gammaproteobacteria | Vibrionales | Vibrionaceae | Vibrio |
| 6SBCC | 0.17 | Proteobacteria | Gammaproteobacteria | Vibrionales | Vibrionaceae | Vibrio |
| 52LBC | 0.07 | Proteobacteria | Gammaproteobacteria | Vibrionales | Vibrionaceae | Vibrio |
| 3LBC | 0.57 | Proteobacteria | Gammaproteobacteria | Vibrionales | Vibrionaceae | Vibrio |
