# Supplementary figures and images for: Spatial and temporal axes impact ecology of the gut microbiome in juvenile European lobster (Homarus gammarus)
Source: ISME J. 2019 Nov 1;14(2):531–43. doi: 10.1038/s41396-019-0546-1 (PMC6976562; doi:10.1038/s41396-019-0546-1)

A

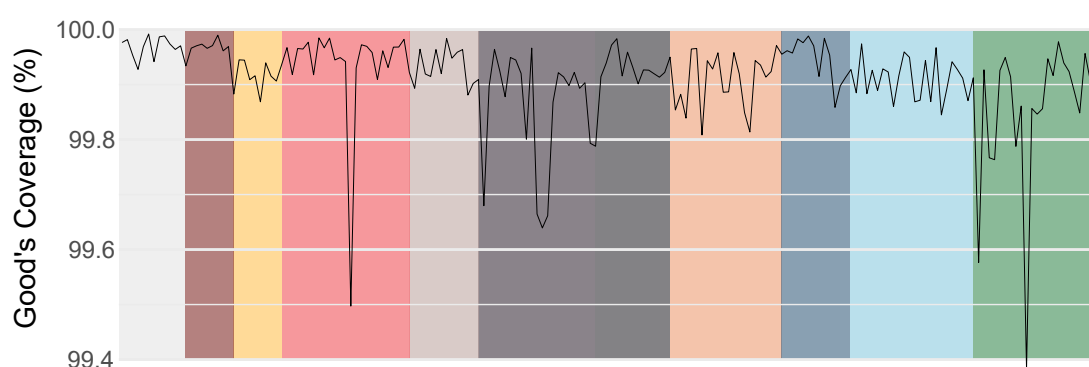

B

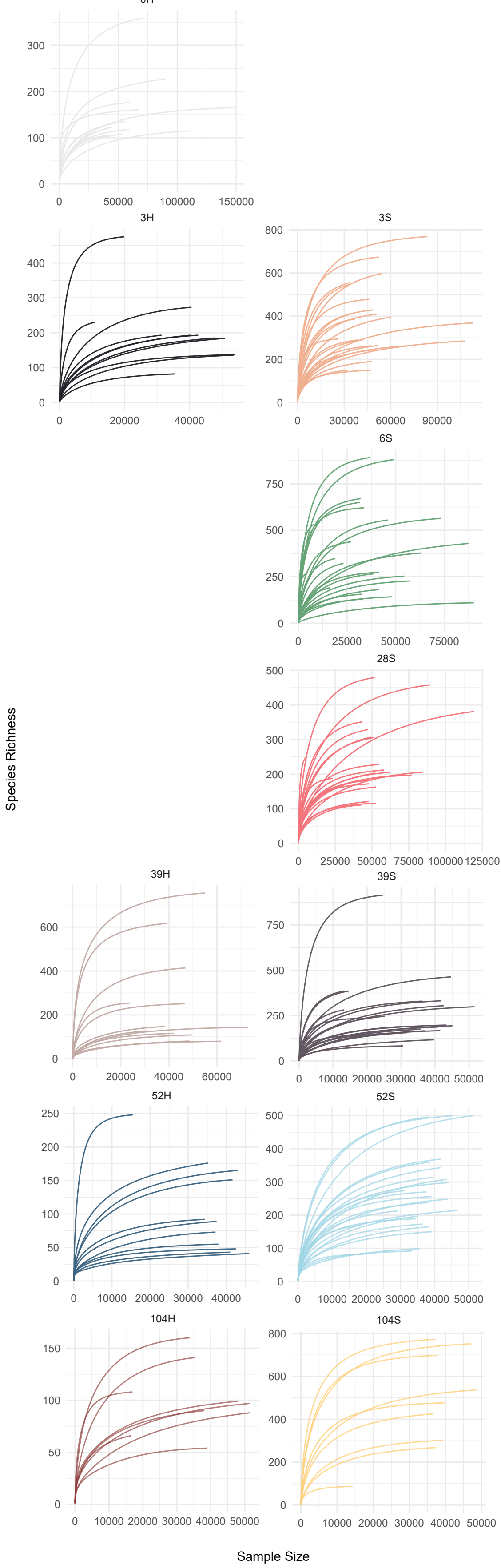

Supplement: Supplementary file 3 — Supplementary Figure 1 [file 41396_2019_546_MOESM3_ESM.pdf]

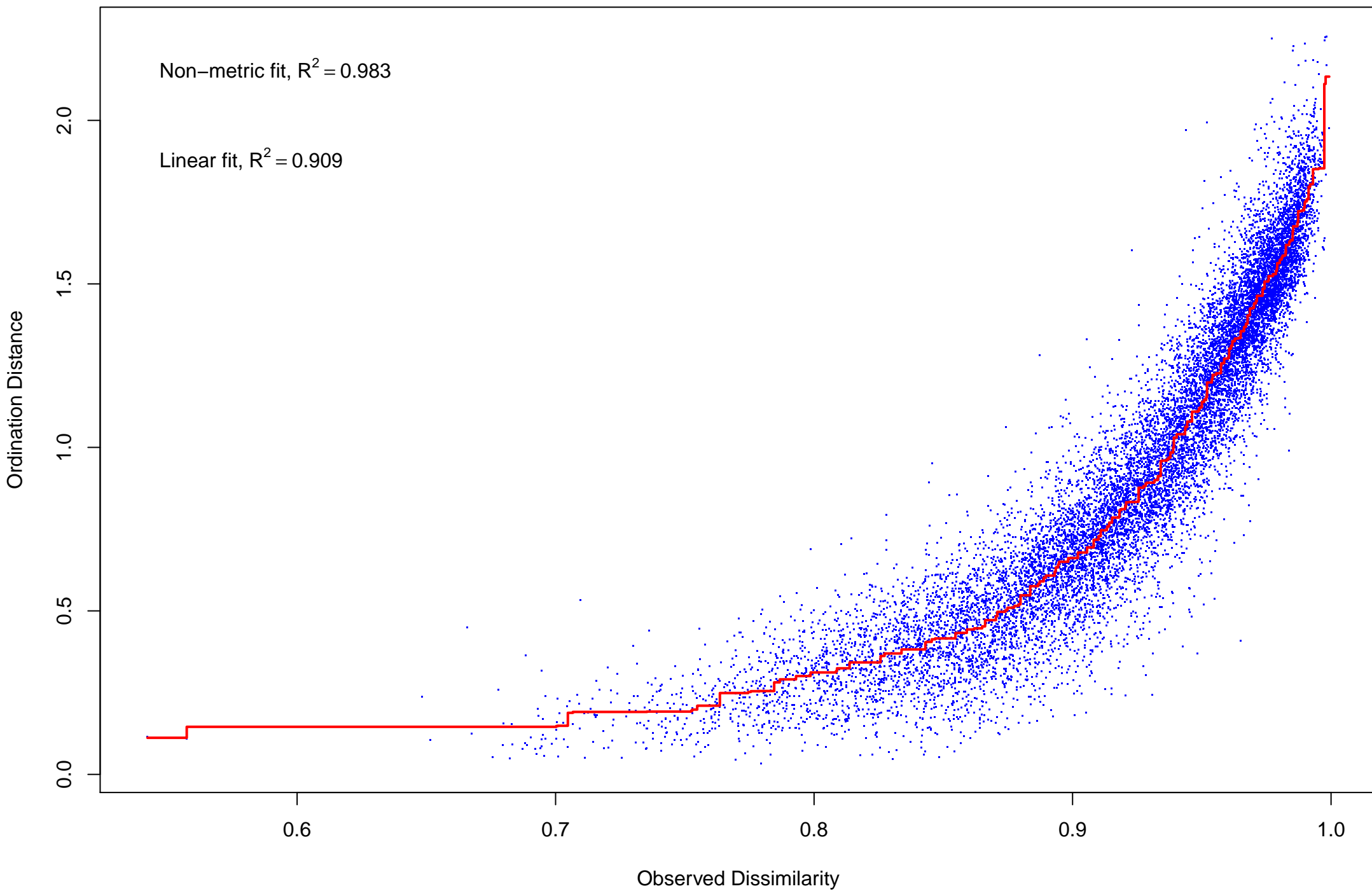

Supplement: Supplementary file 4 — Supplementary Figure 2 [file 41396_2019_546_MOESM4_ESM.pdf]
